# Don’t be Too Strict with Yourself! Rigid Negative Self-Representation in Healthy Subjects Mimics the Neurocognitive Profile of Depression for Autobiographical Memory

**DOI:** 10.3389/fnbeh.2013.00041

**Published:** 2013-05-21

**Authors:** Marco Sperduti, Pénélope Martinelli, Sandrine Kalenzaga, Anne-Dominique Devauchelle, Stéphanie Lion, Caroline Malherbe, Thierry Gallarda, Isabelle Amado, Marie-Odile Krebs, Catherine Oppenheim, Pascale Piolino

**Affiliations:** ^1^Laboratoire Mémoire et Cognition, Institut de Psychologie, Université Paris DescartesBoulogne-Billancourt, France; ^2^INSERM U894, Centre de Psychiatrie et Neurosciences, Université Paris DescartesParis, France; ^3^INSERM U894, Service d’Imagerie, Université Paris Descartes Sorbonne Paris CitéParis, France; ^4^Faculté de Médecine, Centre Hospitalier Sainte-Anne, Service Hospitalier Universitaire, Université Paris DescartesParis, France; ^5^Institut Universitaire de FranceParis, France

**Keywords:** autobiographical memory, depression, executive functions, self, neuroimaging, anterior cingulate cortex, ventro-lateral prefrontal cortex

## Abstract

Autobiographical memory (AM) comprises representation of both specific (episodic) and generic (semantic) personal information. Depression is characterized by a shift from episodic to semantic AM retrieval. According to theoretical models, this process (“overgeneralization”), would be linked to reduced executive resources. Moreover, “overgeneral” memories, accompanied by a negativity bias in depression, lead to a pervasive negative self-representation. As executive functions and AM specificity are also closely intricate among “non-clinical” populations, “overgeneral” memories could result in depressive emotional responses. Consequently, our hypothesis was that the neurocognitive profile of healthy subjects showing a rigid negative self-image would mimic that of patients. Executive functions and self-image were measured and brain activity was recorded, by means of fMRI, during episodic AMs retrieval in young healthy subjects. The results show an inverse correlation, that is, a more rigid and negative self-image produces lower performances in both executive and specific memories. Moreover, higher negative self-image is associated with decreased activity in the left ventro-lateral prefrontal and in the anterior cingulate cortex, repeatedly shown to exhibit altered functioning in depression. Activity in these regions, on the contrary, positively correlates with executive and memory performances, in line with their role in executive functions and AM retrieval. These findings suggest that rigid negative self-image could represent a marker or a vulnerability trait of depression by being linked to reduced executive function efficiency and episodic AM decline. These results are encouraging for psychotherapeutic approaches aimed at cognitive flexibility in depression and other psychiatric disorders.

## Introduction

Autobiographical memory (AM) is the “long term” memory system involved in the retention and retrieval of personal past events. A distinction between episodic AM (EAM) and semantic AM (SAM) has been proposed by several authors (Conway and Pleydell-Pearce, 2000; Conway, 2001; Tulving, 2002; Piolino et al., 2009; Klein and Gangi, 2010). The former refers to memory for unique events situated in time and space and recollected with phenomenological details and a sense of remembering, whereas the latter concerns decontextualized extended or repeated events and self-knowledge such as the name of one’s acquaintances.

Autobiographical memory is impaired in a number of psychiatric disorders including post-traumatic stress disorder (McNally et al., 1994), schizophrenia (Riutort et al., 2003; Harrison and Fowler, 2004; Iqbal et al., 2004), and depression (Brittlebank et al., 1993; Kuyken and Dalgleish, 1995; Brewin et al., 1999; Wessel et al., 2001; Watson et al., 2013). The common pattern of AM impairment in these pathological states is characterized by a specific EAM deficit: patients recalling preferentially “overgeneral” memories (i.e., repeated and extended events) rather than unique episodes with a precise spatio-temporal context.

In depression the “overgenerality” characterizing AM retrieval is accompanied by a particular difficulty in recollecting details, even in the context of specific event retrieval (Lemogne et al., 2006). Interestingly, this lack of specificity has predictive value for the course of depression (Brittlebank et al., 1993; Peeters et al., 2002; Raes et al., 2006; Hermans et al., 2008). Moreover, there is a large body of evidence showing that “overgeneral” AM is not simply a symptom of depression but can be regarded as a trait marker or vulnerability factor for this disease (Williams et al., 2007). Indeed, even in “non-clinical” populations, reduced memory specificity predicts increased emotional reactivity to stressful events (Mackinger et al., 2000; Gibbs and Rude, 2004; Van Minnen et al., 2005; Bryant et al., 2007; for a review, see Raes et al., 2007).

According to the Self-Memory System model (SMS, Conway and Pleydell-Pearce, 2000; Conway, 2005), specific memories are generally accessed through a hierarchical search starting from general events. This process engages executive functions in order to select relevant information and concurrently inhibit competing information. This theoretical model has been supported by numerous studies reporting that the ability to retrieve specific memories is linked to executive processes such as cognitive flexibility, inhibition, updating, shifting, and working memory (Baddeley and Wilson, 1986; Winthorpe and Rabbitt, 1988; Fivush and Nelson, 2004; Matuszewski et al., 2006; Piolino et al., 2007a,b, 2010; Addis et al., 2008; Raes et al., 2010; Ros et al., 2010; Coste et al., 2011).

Thus, “overgeneral” memories may arise when insufficient executive resources cause the premature break of the memory search at higher hierarchical levels. For instance, reduced AM specificity has been shown to be associated with poor performance in verbal fluency in controls (Williams and Dritschel, 1992) and in participants with eating disorders (Dalgleish et al., 2007). In the same vein, Heeren et al. (2009) have reported a parallel improvement of verbal fluency and AM specificity following a mindfulness training. Verbal fluency is sometimes considered as a measure of cognitive flexibility (Heeren et al., 2009), and more generally as a broad measure of executive control (Rosen and Engle, 1997).

Depressed patients show deficits in several executive functions including inhibition (Linville, 1996; MacQueen et al., 2000; Markela-Lerenc et al., 2006; Gohier et al., 2009) and cognitive flexibility (Naismith et al., 2003; Airaksinen et al., 2004; Meiran et al., 2010). Flexibility difficulties have emerged to be among the most prominent cognitive impairment in depression (Austin et al., 2001). Consequently, executive deficits are a central feature of a number of theoretical models of the depressive pathology (Hasher and Zacks, 1979; Ellis and Ashbrook, 1988; Hertel and Rude, 1991; Barrett et al., 2004). Thus, based on Conway and Pleydell-Pearce’s (2000) proposition, the Capture and Rumination, Functional Avoidance, and eXecutive control model (CaRFAX model, Williams et al., 2007) proposes that “overgeneralization” in depression may result from executive impairment, that leads to difficulty in inhibiting inappropriate (i.e., “overgeneral”) memories. Recently, however, Watson et al. (2013) did not find any relationship between verbal fluency and memory specificity in depressed patients.

Nevertheless, contrasting results have been reported concerning the role of executive deficit in depression, with some studies reporting a normalization of performance after recovery from recurrent depression (Beblo et al., 1999; Neu et al., 2001), whereas others reported a persistent impairment after remission (Beats et al., 1996; Reischies and Neu, 2000). Biringer et al. (2005) have shown no significant difference between recovered patients and controls on a composite score measuring global executive functioning, although, in the same group, semantic fluency performance was still below that of the controls. Such results could depend on the particular process tested, it has been proposed that executive deficits may be a risk factor for emotional disorders (Nolen-Hoeksema and Watkins, 2011), particularly for depression (Ingram et al., 2008).

Theoretical considerations (James, 1890; Conway and Pleydell-Pearce, 2000; Gardiner, 2001), as well as behavioral ones (Rogers et al., 1977; Symons and Johnson, 1997) along with neuroimaging findings (Fletcher et al., 1996; Konishi et al., 2000; McDermott et al., 2000; Donaldson et al., 2001; Northoff and Bernpohl, 2004; Henson et al., 2005; Buckner et al., 2008; Sajonz et al., 2010; for reviews, see Cavanna and Trimble, 2006; Legrand and Ruby, 2009) suggest that AM and self-referential processing are intrinsically related. The relationship between self-concept and AM has been illustrated by a recent neuroimaging study reporting a correlation between the degree of certainty in self-evaluation and activity in dorso-medial prefrontal cortex (MPFC) (D’Argembeau et al., 2012). The authors proposed that this correlation may reflect the engagement of processes involved in the retrieval, integration, and evaluation of self-related information allowing the construction of a coherent self-image.

Thus, the categorical nature of “overgeneral” memories may contribute to other forms of “overgeneral” thinking found in depressed patients such as global negative self-judgment and “overgeneralization” in self-evaluation. Indeed, recurrent retrieval of “overgeneral” memories leads to categorical self-descriptors (“I am always boring”) resulting in a retrieval style closely linked to rumination (Watkins and Teasdale, 2001; Raes et al., 2006; Debeer et al., 2011). Thus, depressed patients’ cognition is characterized by negative schemas and a self-focus generating and maintaining a depressed mood and a pessimistic view of the self, the world, and the future (Beck, 1976, 2008; Beck et al., 1979). This negative bias has been associated with executive control impairments (Lo and Allen, 2011; De Lissnyder et al., in press), such as difficulties in inhibiting the processing of negative information (Joormann, 2004; Goeleven et al., 2006; see Gotlib and Joormann, 2010 for review). Such cognitive biases, in particular recurrent negative self-evaluations, are known to be predictive of future depressive symptoms (Carver, 1988; Dent and Teasdale, 1988). Accordingly, Mongrain (1990) demonstrated that high rates of dysfunctional attitudes, characterized by rigid content regarding self-worth leading to poor self-esteem (Mirabel-Sarron et al., 2001), are predictive of subsequent depressive symptoms (Beck, 1967; Beck et al., 1979; Segal and Ingram, 1994).

Cognitive theories posit that information processing and memory retrieval style may constitute a risk factor for the occurrence of depressive episodes. Thus, cognitive dysfunction may be an endophenotype for depression (Hasler et al., 2004). In particular, negative self-schemas (Ingram and Siegle, 2002) and self-evaluations (Carver, 1988; Dent and Teasdale, 1988) as well as rigid attitudes regarding self-judgment (Beck, 1967; Beck et al., 1979; Segal and Ingram, 1994) are known to represent vulnerability factors for depression.

Thus, we hypothesize that a rigid negative self-image in a “non-clinical” population would mimic the neurocognitive profile of depressed patients. To test our hypothesis, we assessed the subjective self-representation of 20 healthy subjects using a standard evaluation. Moreover, we measured different executive functions with standard neuropsychological tests. Then we asked participants to recall specific AMs while recording their brain activity by means of fMRI.

According to the existing literature (Joiner, 2000; Hammen, 2005; O’Brien et al., 2006; Evraire and Dozois, 2011; Morley and Moran, 2011; for review, see Sowislo and Orth, 2012), we expected to find that subjects showing higher negative self-image should show decreased scores on executive functions and, in turn, in the specificity of AMs. Regarding neuroimaging data, we expected to replicate previous findings on AM retrieval reporting activities in a widespread network encompassing fronto-parietal areas, cortical midline regions, and medial temporal structures (for a recent meta-analysis see Martinelli et al., 2012). Moreover, we predicted a significant correlation between the extent of the crystallized negative self-image and the activity in regions frequently reported as dysfunctional in depression (Brody et al., 1999; Mayberg et al., 1999; Drevets and Price, 2005; Murrough et al., 2011), in particular in lateral prefrontal cortex and anterior cingulate cortex (ACC) which are also linked to executive functions and memory retrieval (Ochsner et al., 2004; Kringelbach and Rolls, 2004; Ochsner and Gross, 2005; Niendam et al., 2012).

## Materials and Methods

### Participants

Twenty healthy young volunteers (25–44 years old, mean = 29.2 ± 5.55, 10 women) all right-handed (according to the Edinburgh Handedness Inventory; Oldfield, 1971) and native French speakers participated to the study. All participants gave their informed written consent as required by the local ethic committee (CPP Ile de France 3 n°2687). Exclusion criteria included presence of history of alcohol or substance abuse, head trauma, major diseases affecting brain functions, neuropsychiatric disorders such as clinical depression (tested with the Mini-International Neuropsychiatric Interview, Sheehan et al., 1998). Moreover, all participants were under the cutoff score on the French version of the Beck Depression Inventory (BDI-21, Beck et al., 1988; Bouvard and Cottraux, 1996, cutoff score >14; mean = 2.65 ± 2.53).

### Self-concept assessment

Each subject fulfilled the Tennessee Self-Concept Scale (TSCS, Fitts and Warren, 1996: French version Duval et al., 2007). This scale assesses the multidimensionality of the self over six domains (family, personal, social, moral, physical, academic), and contains 82 descriptive statements (e.g., “I am an honest person”) that have to be rated on a five-point scale (always false, mostly false, partly false/partly true, mostly true, always true) according to how well they match the participant’s personality. Two standard scores were computed: (1) the degree of certainty (TSCS-C) (for a comparable method, see Addis and Tippett, 2004; Naylor and Clare, 2008) was measured through the amount of responses rated “1” (always false) or “5” (always true) and reflects “the degree of certainty about the way one sees oneself, thus reflecting the extent to which a definite sense of identity is expressed” (Naylor and Clare, 2008, p. 595). A more definite sense of self has been shown to reflect a less nuanced and a more crystallized and rigid self-concept (Klein and Gangi, 2010; Martinelli et al., 2013; Picard et al., in press); (2) the total score of the TSCS (TSCS-V) that reflects the global valence of the self (i.e., direction of the self, Addis and Tippett, 2004) and adds up the separate TSCS scores of identity, satisfaction, and behavior. High scores indicate a positive self-concept and higher self-esteem. Finally, a “negative crystallization score” (NCS) was computed by dividing the degree of certainty by the total score (each score being previously transformed into *z* score), so the higher the NCS score the more the self-concept was crystallized and negative.

### Neuropsychological measures

In order to characterize executive and working memory functions, we administered to the participants the following standard tests: the running span (Morris and Jones, 1990; Quinette et al., 2003; total score), the Stroop test (Stroop, 1935; interference score), and trail making test (Reitan, 1958, TMT B-A) to assess updating, inhibition, and shifting functions respectively (Miyake et al., 2000); verbal fluencies (Cardebat et al., 1990, sum of animal and letter P fluency), and digit and visuo-spatial spans (sum of backward and forward spans, Wechsler, 2000) to assess cognitive control and working memory functions. All scores were scaled in the same direction, so that higher scores reflect better performance.

### Neuroimaging procedure

#### Pre-scanning interview

In the pre-scanning interview, exclusion and inclusion criteria were verified by means of a clinical examination and psychometric tests. Then the TSCS and neuropsychological tests were administered to participants. In addition, subjects completed the Taste and Interest Questionnaire (TIQ) that was employed to create personal cues to trigger AM retrieval in the scanning session. The aim of this questionnaire was to collect information in order to create personalized cues for each participant without directly asking for descriptions of past memories to avoid re-encoding memories (Viard et al., 2010; Addis et al., 2011). Participants were informed that the purpose of the questionnaire was to obtain a description of their personality based on information about their main life interests. They had no prior knowledge of the aim of the fMRI task, preventing the possibility for participants of searching for memories linked to their taste and interests between the two sessions. The questionnaire concerned their personal lives from their birth to 5 years ago. It consists of a list of 220 interests including leisure, food, drink, transport, places where they lived, holidays, jobs, studies. For each item, the participants had to answer whether it was personally pertinent or not, rated by 1 and 0 respectively. When an item was pertinent, they had to rate how important (from 0 to 10) and frequent (Frequent/Rare) the activity or interest had been in their life. An activity or interest was used as a cue for episodic AM retrieval if it was pertinent, important (>5), and rare. Twenty-four cues were created for each subject. Examples of activity assessed in the TIQ and the procedure to create cues is illustrated in the Table A1 in Appendix.

#### Episodic autobiographical memory task

The participants were first invited to take part in a training session before the fMRI scanning. Participants received detailed explanations on the nature of the task and participated in a brief simulation of the experiment on a laptop. They were instructed to recall EAMs elicited by the cues and to press a button when a memory was recalled. EAMs were defined as memories of a single event that occurred at a specific time and place, of short duration, lasting less than 24 h. Participants were instructed to mentally relive personal episodes prompted by cues and to recollect affective and perceptual details (such as time, location, perceptions, feelings, scenery, and people present in the scene) (e.g.: “a unique memory linked to a trip in Italy”). After instructions, participants were trained on three trials with the experimenter providing feedback concerning the pertinence of the responses. The cues used for training were different from those used during the scanning session.

#### Scanning session

During fMRI recording, cues were visually presented in white font on a black background projected on a screen viewed by means of a mirror incorporated into the head-coil. E-Prime software (Psychology Software Tools, Inc., Pittsburgh, PA, USA) in combination with an Integrated Functional Imaging System (IFIS) was used for the presentation and timing of stimuli and collection of responses. Responses were made on an MR-compatible two-buttons box. Participants completed four functional scans in a single session. Each functional scan was composed of six items. Each trial lasted 26 s with the following time-course: the cue was presented for 5 s, followed by a white cross at the center of the screen for 19 s, then the cross turned red for 3 s informing the participants of the end of the present trial and the arrival of the next one. Participants were instructed to press a button as soon as they accessed a memory.

#### Post scan interview

Participants were asked to recall again each EAM retrieved in the scanner in order to check that memories met minimal criteria of specificity (single events, situated in time and place, lasting less than 24 h, e.g., “the day of the visit of the exhibition ‘The man on the moon’ in the Palace of Tokyo museum in Paris, in August 2009”). The subsequent analyses were performed only on memories that met all the above mentioned criteria.

Episodic AMs were rated for specificity on standard scales (Levine et al., 2002; Piolino et al., 2009). More precisely, the presence of specific spatial and temporal details, and other contextual and phenomenological details in each evocation was noted (one point by type of detail, max. 4; e.g., “I remember my visit in the Palace of Tokyo as if I was still there, being together with Chiara in a room of the exhibition in the first floor in the dark to see the TV reports and talking with other visitors…, it was 6:00 p.m., after then we settled down in the restaurant of the outdoor museum in front of the Seine…”). We computed for each participant a global ratio of specificity (EPI score) totaling up the sum of spatio-temporal, other contextual and phenomenological details, divided by the number of EAM.

### fMRI method

#### MRI data acquisition

All data were acquired with a 3 T scanner (MR 750, General Electric Healthcare, Little Chalfont, UK). The anatomical scan used an inversion recovery 3-D T1-weighted gradient-echo sequence images (TE = 4.3 ms, TR = 11.2 ms, TI = 400 ms, matrix = 384 × 384, slice thickness = 1.2 mm). Functional images were acquired using a gradient-echo echoplanar (EPI) sequence (TE = 30 ms, TR = 2000 ms, flip angle = 90°, matrix = 64 × 64, slice thickness = 3 mm, 42 contiguous sections). The first four volumes of each functional run were discarded in order to allow longitudinal magnetization to approach equilibrium.

#### Pre-processing of fMRI data

All data were processed using SPM5 software (Statistical Parametric Mapping 5, Welcome Dept. Cognitive Neurology, UK; www.fil.ion.ucl.ac.uk/spm). Standard pre-processing procedures were applied to MRI data. EPI volumes were corrected for slice timing, realigned to the first image, co-registered with the high-resolution T_1_-weighted image and normalized into the Montreal Neurological Institute (MNI) template. Finally, the normalized EPI volumes were smoothed using an isotropic Gaussian kernel filter of 5 mm full-width half-maximum.

#### First level analysis of fMRI data

Only correct trials were used for the subsequent analyses. A trial was considered as correct if (1) the participant had pressed the button during the trial (indicating retrieval) and (2) the description of the memory during the debriefing corresponded to EAM (see above). Memory retrieval (i.e., access or strategic research phase) was modeled by convolving the time period between cue presentation and subjects’ response with the hemodynamic response function (HRF). For each subject, General Linear Model was used to estimate the parameters of interest. Parameters of movement were also included in the model as regressors of no interest. A whole brain *t*-test was computed to estimate the contrast of interest for each subject: EAM vs. rest. Then, contrasts for each individual were used for second-level analyses.

#### Second-level analysis of fMRI data

We computed a whole brain *t*-test using first level contrasts for each subject. An activation map resulting from this analysis was then used to mask subsequent correlation analysis. The rationale of this choice was that we were only interested in correlations in areas showing a significant activation. Threshold for the whole brain *t*-test was fixed at *p* < 0.01 corrected for multiple comparison using the false discovery rate (FDR) with an extended threshold of *k* = 20.

#### Correlations

We computed correlations between signal change in regions showing a significant activity at the group level and the NCS using the multiple regression model in SPM in which we entered contrast images as well as the NCS for each subject as a covariate. The threshold for this analysis was fixed at *p* < 0.01 (uncorrected) with an extended threshold of *k* = 10. Then we extracted percentage signal change of clusters showing a significant correlation using Marsbar toolbox (Brett et al., 2002) and calculated correlations between signal change and the executive and EAM scores outside SPM using STATISTICA7©.

## Results

### Behavioral results

Participants showed a high percentage of correct trials (CR, mean 87.85 ± 7.70) and a rapid response time (RT, mean 2.28 ± 0.94 s). NCS correlated negatively with inhibition, verbal fluency, and working memory performances. A trend for a negative correlation between NCS and the episodic score was found (*r* = −0.44, *p* = 0.054). Interestingly, the two basic scores of the TSCS, certainty (TSCS-C) and valence (TSCS-V) of self-concept, did not singularly correlate with executive functions and the episodic score. The episodic score correlated positively with performance on executive functions, namely inhibition, shifting (TMT B-A), verbal fluency, and working memory. For detailed results see Table 1.

**Table 1:** **Correlations between self-concept, episodic and neuropsychological scores**.

	NCS	TSCS-C	TSCS-V	EPI	FLU	INHIB	TMT B-A	R-SPAN	WM	CR	RT
NCS		0.040	−0.365	−0.438	**−0.626**	**−0.582**	−0.271	−0.203	**−0.449**	0.149	0.011
*p*		0.869	0.114	0.054	**0.003**	**0.007**	0.248	0.391	**0.047**	0.532	0.965
TSCS-C	0.040		**0.748**	−0.118	−0.227	−0.051	0.120	0.114	−0.058	0.086	−0.321
*p*	0.869		**0.000**	0.622	0.337	0.832	0.613	0.632	0.809	0.719	0.168
TSCS-V	−0.365	**0.748**		0.040	0.207	0.194	0.202	0.369	0.346	−0.020	−0.094
*p*	0.114	**0.000**		0.867	0.381	0.412	0.394	0.110	0.135	0.934	0.692
EPI	−0.438	−0.118	0.040		**0.523**	**0.545**	**0.451**	0.411	**0.668**	0.363	0.076
*p*	0.054	0.622	0.867		**0.018**	**0.013**	**0.046**	0.072	**0.001**	0.115	0.750
FLU	−**0.626**	−0.227	0.207	**0.523**		**0.478**	**0.536**	**0.492**	**0.596**	0.264	−0.008
*p*	**0.003**	0.337	0.381	**0.018**		**0.033**	**0.015**	**0.027**	**0.006**	0.260	0.975
INHIB	−**0.582**	−0.051	0.194	**0.545**	**0.478**		−0.275	**0.498**	**0.581**	−0.074	0.088
*p*	**0.007**	0.832	0.412	**0.013**	**0.033**		0.241	**0.025**	**0.007**	0.755	0.712
TMT B-A	−0.271	0.120	0.202	**0.451**	**0.536**	−0.275		0.429	0.417	**0.460**	−0.209
*p*	0.248	0.613	0.394	**0.046**	**0.015**	0.241		0.059	0.068	**0.041**	0.377
R-SPAN	−0.203	0.114	0.369	0.411	**0.492**	**0.498**	0.429		**0.689**	0.100	0.174
*p*	0.391	0.632	0.110	0.072	**0.027**	**0.025**	0.059		**0.001**	0.677	0.462
WM	−**0.449**	−0.058	0.346	**0.668**	**0.596**	**0.581**	0.417	**0.689**		0.134	0.435
*p*	**0.047**	0.809	0.135	**0.001**	**0.006**	**0.007**	0.068	**0.001**		0.573	0.055
CR	0.149	0.086	−0.020	0.363	0.264	−0.074	**0.460**	0.100	0.134		−0.189
*p*	0.532	0.719	0.934	0.115	0.260	0.755	**0.041**	0.677	0.573		0.425
RT	0.011	−0.321	−0.094	0.076	−0.008	0.088	−0.209	0.174	0.435	−0.189	
*p*	0.965	0.168	0.692	0.750	0.975	0.712	0.377	0.462	0.055	0.425	

*NCS, negative crystallization score; TSCS-C, TSCS, certainty score; TSCS-V, TSCS, valence score; EPI, episodic score of EAM; FLU, verbal fluency score; INHIB, interference score Stroop, inhibition; TMTB-A, trail making test B-A score, shifting; R-SPAN, running span, updating; WM, working memory score, digit and visuo-spatial spans; CR, correct responses; RT, response time. Correlations written in bold font are significant (*p* < 0.05 to *p* < 0.001)*.

### fMRI results

#### Activation during EAM retrieval

**Figure 1:**
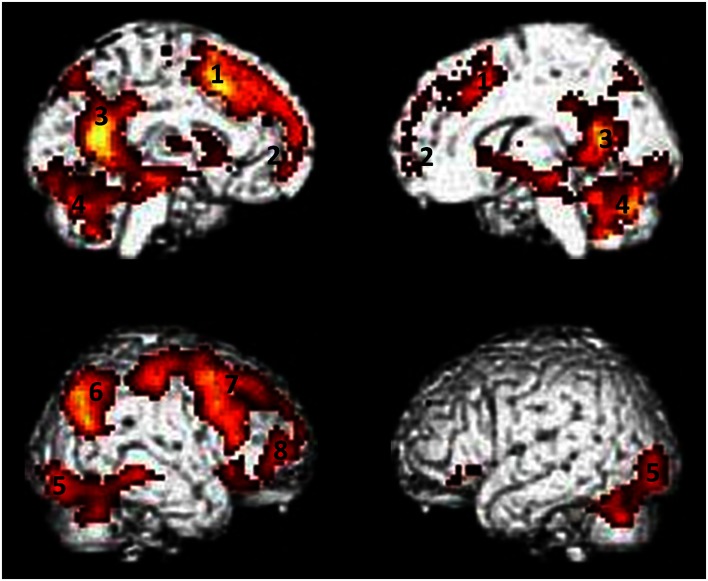
**Areas showing activity during retrieval of episodic autobiographical memory**. (1) Dorsal anterior cingulate cortex (dACC), (2) medial prefrontal cortex (MPFC), (3) posterior cingulate cortex/precuneus (PCC/precuneus), (4) cerebellum, (5) occipital cortex, (6) posterior parietal cortex (PPC), (7) dorso-lateral prefrontal cortex (dLPFC), (8) ventro-lateral prefrontal cortex (vLPFC). Results are superimposed to a single subject rendering and are significant at a threshold of *p* < 0.01 corrected for multiple comparison using the false discovery rate (FDR) with an extended threshold of *k* = 20.

We reported activations in several clusters encompassing lateral (mainly on the left side) and medial frontal regions and posterior medial regions. In particular we found activations in cortical midline structures comprising MPFC, ACC, posterior cingulate (PCC), and precuneus. Moreover insula, cerebellum, inferior parietal, and occipital regions as well as lateral and medial temporal regions comprising the hippocampus were found (Figure 1). The list of local activation maxima is reported on Table 2.

**Table 2:** **List of regions activated during memory retrieval**.

Lobe	Labels	BA	*t*	MNI		
				*x*	*y*	*z*
Frontal	ACC	32	13.91	−6	15	45
	Mid. front.	6	11.81	−39	9	54
	Prec. gyr.	4	8.36	−36	−27	54
	SMA	6	8.04	−6	18	63
	Mid. front.	8	7.81	−24	27	51
	vLPFC	44	7.71	−51	12	36
	dLPFC	9	7.59	−18	36	45
	vLPFC	45	6.53	−54	24	15
	dLPFC	9	6.37	−9	54	36
	vMPFC	11	6.34	−9	60	−6
	Mid. front.	10	6.26	−33	48	12
	Mid. CC	23	6.09	0	−36	33
	Prec. gyr.	6	5.98	−33	−18	63
	vLPFC	47	5.59	−36	45	3
	vLPFC	47	4.61	30	27	0
Parietal	PCC	23	12.28	−6	−57	18
	Pre. cun.	7	11.53	6	−57	−48
	Inf. par.	7	10.37	−36	−72	42
	Ang. gyr.	39	9.98	−48	−69	30
	Inf. par.	40	8.72	−39	−33	48
	Sup. par.	7	7.54	−30	−69	51
	Postc. gyr.	3	7.31	−45	−27	51
Temporal	Fus. gyr.	37	10.3	−27	−36	−18
	Fus. gyr.	37	8.43	30	−57	−27
	Inf. temp.	20	6.56	−54	−39	−12
	Hipp.		5.54	−21	−18	−15
	pHipp.		4.94	24	−18	−21
Occipital	Calcarine	17	13.48	−9	−60	12
	Lingual gyr.	17	12.18	12	−54	9
	Calcarine	18	8.55	9	−72	−30
	Inf. occ.	19	8.01	−39	−87	−6
	Inf. occ.	18	6.29	−30	−90	−6
	Inf. occ.	19	6.21	39	−75	−18
Other	Cerebellum		6.65	0	−66	−30
	Insula		5.95	−30	24	−3

*BA, Brodmann area; ACC, anterior cingulate cortex; Mid. front., middle frontal gyrus; Prec. gyr., precentral gyrus; SMA, supplementary motor area; vLPFC, ventro-lateral prefrontal cortex; dLPFC, dorso-lateral prefrontal cortex; vMPFC, ventro-medial prefrontal cortex; Mid. CC, middle cingulate cortex; PCC, posterior cingulate cortex; Pre. cun., precuneus; Inf. par., inferior parietal gyrus; Ang. gyr., angular gyrus; Sup. par., superior parietal gyrus; Postc. gyr., postcentral gyrus; Fus. gyr., fusiform gyrus; Inf. temp., inferior temporal gyrus; Hipp., hippocampus; pHipp, parahippocampus; Lingual gyr., lingual gyrus; Inf. occ., inferior occipital gyrus*.

#### Correlation between brain activations, neuropsychological, and EAM scores

**Figure 2:**
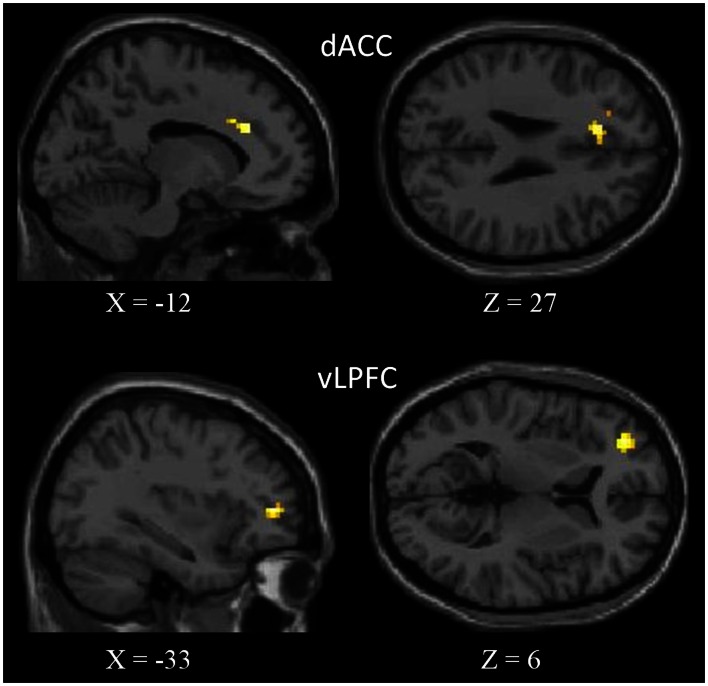
**Areas showing negative correlation with the NCS**. In the upper part of figure, the dorsal anterior cingulate cortex (dACC), and in the bottom, the ventro-lateral prefrontal cortex (vLPFC). Results are superimposed to a single subject T1-weighted image normalized to the MNI stereotaxic space. Results are significant at a threshold of *p* < 0.01 (uncorrected) with an extended threshold of *k* = 10.

We observed negative correlations between the NCS and the dorsal ACC (dACC) and the ventro-lateral prefrontal cortex (vLPFC) in the left side (Figure 2). See Table 3 for peaks coordinates. A positive correlation was reported between verbal fluency and both regions, whereas only the dACC showed a significant correlation with inhibition performance. Moreover, for correlations between activity in these regions and the other scores of interest we found a positive correlation with episodic scores. The basic scores of the TSCS did not show significant correlations with the other variables. For detailed results see Table 4.

**Table 3:** **List of regions showing a correlation with the CNS**.

Lobe	Labels	BA	*t*	MNI		
				*x*	*y*	*z*
Frontal	vLPFC	47	4.24	−33	45	6
	dACC	32	3.76	−12	27	27

*BA, Brodmann area; dACC, dorsal anterior cingulate cortex; vLPFC, ventro-lateral prefrontal cortex*.

**Table 4:** **Correlations between brain activity self-concept, episodic and neuropsychological scores**.

	NCS	TSCS-C	TSCS-V	EPI	FLU	INHIB	TMT B-A	R-SPAN	WM
dACC	−**0.717**	−0.001	0.153	**0.630**	**0.504**	**0.587**	0.285	0.114	0.330
*p*	**0.000**	0.996	0.520	**0.003**	**0.024**	**0.007**	0.224	0.634	0.155
vLPFC	−**0.632**	0.156	0.233	**0.591**	**0.538**	0.311	0.332	0.127	0.333
*p*	**0.003**	0.511	0.324	**0.006**	**0.015**	0.182	0.152	0.593	0.151

*NCS, negative crystallization score; TSCS-C, TSCS, certainty score; TSCS-V, TSCS, valence score; EPI, episodic score of EAM; FLU, verbal fluency score; INHIB, interference score Stroop, inhibition; TMTB-A, trail making test B-A score, shifting; R-SPAN, running span, updating; WM, working memory score, digit and visuo-spatial spans; dACC, dorsal anterior cingulate cortex; vLPFC, ventro-lateral prefrontal cortex. Correlations written in bold font are significant (*p* < 0.05 to *p* < 0.001)*.

A series of partial correlations (Bravais–Pearson) were calculated between the NCS, the episodic score, and activity in the dACC and vLPFC, separately controlling for inhibition, fluency, and working memory scores. When controlling for inhibition, fluency, or working memory performance the correlation between NCS and the episodic score disappeared. Moreover, when controlling for the fluency score, the correlations between the NCS and the vLPFC, and between the episodic score and the same region become marginally significant. See Table 5 for detailed results.

**Table 5:** **Partial correlations between brain activity, self-concept, and episodic score, controlling for executive functions performances**.

	NCS	EPI	dACC	vLPFC
**INHIB**				
NCS		−0.18	−**0.57**	−**0.58**
*p*		0.469	**0.011**	**0.009**
EPI	−0.18		**0.46**	**0.53**
*p*	0.469		**0.049**	**0.020**
dACC	−**0.57**	**0.46**		**0.77**
*p*	**0.011**	**0.049**		**0.000**
vLPFC	−**0.58**	**0.53**	**0.77**	
*p*	**0.009**	**0.020**	**0.000**	
**FLU**				
NCS		−0.17	−**0.60**	−0.45
*p*		0.499	**0.007**	0.053
EPI	−0.17		**0.50**	0.43
*p*	0.499		**0.030**	0.066
dACC	−**0.60**	**0.50**		**0.70**
*p*	**0.007**	**0.030**		**0.001**
vLPFC	−0.45	0.43	**0.70**	
*p*	0.053	0.066	**0.001**	
**WM**				
NCS		−0.21	−**0.67**	−**0.57**
*p*		0.395	**0.002**	**0.010**
EPI	−0.21		**0.58**	**0.52**
*p*	0.395		**0.009**	**0.021**
dACC	−**0.67**	**0.58**		**0.75**
*p*	**0.002**	**0.009**		**0.000**
vLPFC	−**0.57**	**0.52**	**0.75**	
*p*	**0.010**	**0.021**	**0.000**	

*NCS, negative crystallization score; EPI, episodic score of EAM; INHIB, interference score Stroop, inhibition; FLU, verbal fluency score; WM, working memory score; dACC, dorsal anterior cingulate cortex; vLPFC, ventro-lateral prefrontal cortex. Correlations written in bold font are significant (*p* < 0.05 to *p* < 0.001)*.

## Discussion

In the present study we assessed, through standard tests, self-concept, executive functions’ profile as well as brain activations during an EAM retrieval task in a group of young healthy subjects. In line with our hypotheses we reported that participants with a rigid negative self-representation tend to retrieve less detailed memories and show poorer performance on executive scores, in particular on inhibition, verbal fluency, and working memory. Interestingly, the valence and the certainty of self-representation taken alone did not seem to be linked either with executive functions, or with EAM performance. This result suggests that a negative self-representation accompanied by a flexible cognitive style would not necessarily lead to “depressive-like” cognitive functioning, and that a rigid self schema would not be inadaptative if not centered on negative content (see Martinelli et al., 2012). Moreover, two regions that were activated in the access or strategic research phase of EAM retrieval, the dACC and the left vLPFC, showed a negative correlation with the NCS, a positive correlation with verbal fluency, and a positive correlation with the episodic scores. Finally, only activity in dACC correlated significantly with inhibition.

Our results are coherent with models of AM retrieval assigning a central role to executive functions in the hierarchical search of episodic details (Baddeley and Wilson, 1986; Conway and Fthenaki, 2000; Conway and Pleydell-Pearce, 2000). Moreover, the negative correlation between executive and the NCS scores is in agreement with previous proposals of executive dysfunction as a trait marker or risk factor for depression (Hasler et al., 2004). Overall, our results suggest that executive functions could have a central role in both inefficient search mechanisms during EAM retrieval and in the construction of a rigid or schematic self-representation, concerning, above all, negative content. Of particular interest is the fact that the marginally significant correlation we found between the NCS and episodic details was removed when controlling for executive functions. Thus, executive functions may mediate the relationship between the NCS and low episodic score. This is in line with the CaRFAX model that assigns a central role to executive deficits in reduced AM specificity in depression (Williams et al., 2007). Also, our neuroimaging results indicated that activity in dACC and vLPFC that were engaged in EAM retrieval, negatively correlated with the NCS and, in turn, positively correlated with executive functions and episodic details, suggesting shared neurocognitive processes.

The ACC has been divided into a dorsal “cognitive” and a rostral “emotional” component (Bush et al., 1998; Whalen et al., 1998; Etkin et al., 2006). The dACC is commonly reported to be recruited during tasks eliciting cognitive control, conflict resolution, and error monitoring (Bush et al., 2000; Beckmann et al., 2009). It has been found to be activated, together with other fronto-parietal regions, across diverse executive functions such as flexibility, inhibition, shifting, and working memory (Hedden and Gabrieli, 2010; Niendam et al., 2012). Regarding inhibition specifically, the positive correlation we found between dACC activity and the interference score corroborates previous findings reporting specific activity in the dACC during Stroop tasks (Bush et al., 1998). Moreover, ACC activity elicited during a Stroop task has been shown to be lower after a negative mood induction (Nixon et al., 2012).

Thus, dACC can be seen as supporting superordinate cognitive control processes (Niendam et al., 2012). This is in line with its role in EAM retrieval and with the pattern of correlations reported in the present study. Indeed, as mentioned above, during memory search, executive functions are supposed to be recruited to select relevant information and concurrently inhibit competing information.

Moreover, in healthy subjects, the dACC is known to exert an inhibitory influence over the limbic system that is devoted to emotional processing (Bush et al., 2000; Shafritz et al., 2006). In individuals with depression, hypo-activation in dACC is assumed to disrupt this inhibitory control leading to the attentional bias for negative information (Greicius et al., 2007). Indeed, in a task requiring participants to disengage attention from negative irrelevant material, depressed patients showed increased activity of the dACC, suggesting a greater cognitive and neural resources requirement during controlled emotional processing (Foland-Ross et al., 2013). Thus, the lower activity of dACC in subjects with greater NCS scores and the concurrent positive correlation between its activity and the inhibition score seems to mimic the neural profile associated with impaired emotional control in depression. This assumption appears quite relevant considering that the Stroop task has been shown to activate the dACC in healthy controls but not in subjects with mood-disorders (George et al., 1997).

Convincing evidence suggests that lateral prefrontal cortex is involved in high-order control processes regulating cognition and behavior (Miller, 2000; Miller et al., 2002; Petrides, 2005). Within prefrontal cortex, a dorso-ventral functional specialization has been proposed. The dorso-lateral prefrontal cortex would be engaged in on-line monitoring and manipulation of information in working memory, whereas the vLPFC would underpin active selection, comparison, and judgment of information held in short and long term memory (Petrides, 1995, 2002, 2005). Concerning memory retrieval, the same author reported that vLPFC, corresponding to BA 45 and 47, would be essential when active strategic retrieval of memories is at stake, but not during automatic retrieval. More recently, Badre and Wagner (2007) proposed a further subdivision of the vLPFC into the anterior vLPFC, corresponding to BA 47, and the mid-vLPFC, composed by BA 45. They reported evidence for a two-process account of controlled memory retrieval mechanisms implemented in the vLPFC with the anterior portion engaged in strategic processes and top down facilitation of relevant information and the mid-vLPFC that would be especially in charge of post retrieval selection of relevant information between competing representations. This account of the anterior vLPFC involvement in effortful strategic memory retrieval fits well with its activation in our task and the correlation found with performance on verbal fluency tasks.

Besides its strategic role in memory retrieval, vLPFC is also known to modulate emotional responses of the amygdala through an attentional biasing mechanism (Wager et al., 2008). Moreover, vLPFC is frequently altered in depression at both the functional (Brody et al., 1999; Mayberg et al., 1999) and structural levels (mainly BA 47, Drevets and Price, 2005). These changes may participate in explaining the depression-related negative bias. Indeed, there is evidence of attenuated neural response in the vLPFC of depressed patients when responding to targets that were preceded by sad distracters (Wang et al., 2008; Dichter et al., 2009). Moreover, rumination on bad feelings and past experiences is maintained in depressed patients by an impaired cognitive control mechanism associated with the hypo-activation of the left prefrontal regions, in particular of the vLPFC (Ochsner et al., 2004; Ray et al., 2005; Gotlib and Hamilton, 2008).

Based on the aforementioned literature on abnormalities in emotional processing in depression, Murrough et al. (2011) proposed a model suggesting that depression-related functional changes are characterized by an imbalance between the cognitive control, implemented in the PFC, and the emotional system, based on limbic structures. In other words, the under-activity of the former regions is thought to mediate executive impairment and to contribute to explaining the failure of cognitive control on emotion in depression.

Interestingly, Beevers et al. (2010) reported that patients with a mild to moderate depression experienced difficulty recruiting regions involved in cognitive control, notably vLPFC, when processing emotional information, whereas activity of cerebral regions that typically subserve emotional experience *per se*, such as amygdala and orbital PFC, were not associated with depressive symptoms. The authors concluded that more severe forms of depression may be necessary before neural activity in these emotional processing regions would be attained (Siegle et al., 2007; Hamilton and Gotlib, 2008).

In summary, according to the aforementioned literature, we propose that the neurocognitive profile of people with a negative crystallized self-representation would mimic that of mildly to moderately depressed patients. In particular, the negative rigid self-representation might result from diminished executive functions resources that, in turn, could affect EAM. This cognitive-profile pattern would be expressed at the neural level as an inefficient recruitment of prefrontal regions normally involved in cognitive control.

Our findings could have a potential impact on research on neurocognitive markers of depression and are encouraging for a psychotherapeutic approach promoting cognitive flexibility, such as novel cognitive behavioral therapies integrating mindfulness practices. Indeed, mindfulness meditation has been shown to produce structural and functional changes in the lateral PFC and in the ACC (Chiesa and Serretti, 2010; Tang et al., 2012), and to improve autobiographical specificity in formerly depressed patients (Williams et al., 2000) and in healthy subjects (Heeren et al., 2009). Moreover, in the latter study improved AM was correlated with enhanced executive functions.

In conclusion, we showed in healthy young participants, that the degree of crystallized negative self-representation mimics the cognitive profile reported in depression concerning executive functions and AM, and that this pattern could be mediated by an inefficient recruitment of prefrontal structures involved in cognitive control of emotional response.

## Conflict of Interest Statement

The authors declare that the research was conducted in the absence of any commercial or financial relationships that could be construed as a potential conflict of interest.

## Appendix

**Table A1:** **Excerpt of the Taste and Interest Questionnaire (TIQ)**.

Activities	Pertinence	Importance	Frequency
**TRAVELS**			
France	1	7	F
Abroad	1	8	F
**Asia**	**1**	**9**	**R**
North America	0		
South America	0		
**Africa**	**1**	**9**	**R**
North Europe	1	6	F
South Europe	1	8	F
East Europe	0		
**PHYSICAL ACTIVITIES**			
Sports (general)	1	8	F
Team sports	0		
Water sports	0		
Tennis	1	4	R
**Golf**	**1**	**8**	**R**
Athletics	0		
**Relaxation**	**1**	**9**	**R**
Equitation	1	10	F
Dance	0		

*Activities were selected as a cue for autobiographical recall only if they were pertinent, important (>5), and rare. Items marked in bold are examples of activities selected according the above mentioned criteria*.
